# Real-time interactive data mining for chemical imaging information: application to automated histopathology

**DOI:** 10.1186/1471-2105-14-156

**Published:** 2013-05-08

**Authors:** David Mayerich, Michael Walsh, Matthew Schulmerich, Rohit Bhargava

**Affiliations:** 1 Beckman Institute for Advanced Science and Technology, University of Illinois at Urbana–Champaign, Urbana, IL, USA; 2 Departmet of Pathology, University of Illinois at Chicago, Chicago, IL, USA; 3 Department of Bioengineering, University of Illinois at Urbana–Champaign, Urbana, IL, USA; 4 Department of Mechanical Science and Engineering, University of Illinois at Urbana–Champaign, Urbana, IL, USA; 5 Micro and Nanotechnology Laboratory, University of Illinois at Urbana–Champaign, Urbana, IL, USA; 6 University of Illinois Cancer Center, Urbana, IL, USA

## Abstract

**Background:**

Vibrational spectroscopic imaging is now used in several fields to acquire molecular information from microscopically heterogeneous systems. Recent advances have led to promising applications in tissue analysis for cancer research, where chemical information can be used to identify cell types and disease. However, recorded spectra are affected by the morphology of the tissue sample, making identification of chemical structures difficult.

**Results:**

Extracting features that can be used to classify tissue is a cumbersome manual process which limits this technology from wide applicability. In this paper, we describe a method for interactive data mining of spectral features using GPU-based manipulation of the spectral distribution.

**Conclusions:**

This allows researchers to quickly identify chemical features corresponding to cell type. These features are then applied to tissue samples in order to visualize the chemical composition of the tissue without the use of chemical stains.

## Background

Vibrational spectroscopic imaging, or chemical imaging, data is composed of a series of absorption or scattering measurements taken across the electromagnetic spectrum. Materials exhibit a characteristic spectral signature that is indicative of their molecular composition. The speed and versatility of vibrational techniques offer the most potential for label-free microscopy by providing micron-scale resolution and significant molecular detail.

In this paper we focus on mid-infrared spectroscopic imaging [1], which is a form of vibrational spectroscopy with a data rate that is currently viable for clinical applications. We will also show that our methods are applicable to other techniques, such as Raman spectroscopy, which show promise for future *in vivo* imaging. Recent advances in optical detector technology allow the rapid acquisition of spectral signals that are spatially specific, producing multidimensional images that can be extensive in size (several tens of gigabytes). These techniques have shown promise in biomedicine for cell type classification [2,3] and cancer analysis [4]. However, the interpretation of a spectral signature is a complex task, requiring a significant amount of pre-processing before identifying spectral features that correspond to chemical information.

While the acquisition of data is relatively rapid, there are limited options at present to assist researchers in visualizing data. In this paper, we propose a method for interactively exploring the chemical composition of a tissue sample. The proposed software allows a user to quickly identify spectral features corresponding to specific tissue types within a sample. The results are then applied to other samples in order to identify tissue types without the use of histological labels. This allows us to overcome several disadvantages of current histology methods by using quantitative information that is collected non-destructively. This makes our methods repeatable and provides an array of useful quantitative information that can be used by a pathologist to aid in diagnosis.

In Section ‘Mid-infrared spectroscopy’, we provide an overview of mid-infrared spectroscopic imaging. Section ‘Morphological effects’ describes the coupling between tissue morphology and chemistry, which makes the characterization of tissue difficult. Our method and implementation details are described in Section ‘Methods’ and Section ‘Visualization’. Section ‘Results and discussion’ provides validation using chemical phantoms and demonstrates our results on breast cancer biopsies.

Our *SpecVis* software is open-source and 32-bit Windows binaries available online (http://www.chemimage.illinois.edu/software).

## Mid-infrared spectroscopy

**Figure 1:**
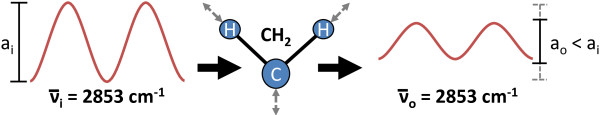
**Mid-infrared spectroscopy measures the light absorbed due to resonance with specific vibrational modes in a molecule.** When transmitted through a material containing a CH_2_ functional group, as shown in this example, the output intensity is lower than the incident light when ${\bar{\nu }}_{i}=$ 2853 cm ^−1^.

Mid-infrared spectroscopy is used to identify molecular content in a sample by determining the fraction of incident light absorbed. Broadband mid-infrared electromagnetic radiation is transmitted through the sample and the absorbed light at each wavelength is quantified (Figure 1). The most common technique for measuring absorption spectra is Fourier Transform Infrared (FT-IR) Spectroscopy [5], which uses an interferometer to encode optical frequencies in time. The recorded data is then decoded using a Fourier transform. The resulting data consist of a spectrum representing the intensity of light transmitted as a function of frequency $\bar{\nu }$. The frequency domain is generally recorded in units of *wavenumber* (cm ^−1^). The absorbance at each wavelength is computed using

(1) $$A(\bar{\nu })=-\text{lo}g_{10}\left(\frac{I(\bar{\nu })}{I_{0}(\bar{\nu })}\right)$$

where *A* is the absorbance, *I* is detected light through the sample, and *I*_0_ is the light detected without the sample present. Molecular bonds are identified by their characteristic pattern where $A(\bar{\nu })$ is non-zero.

Recent advances in FT-IR spectrometry allow the use of focal plane arrays (FPAs) for acquiring spatially-resolved mid-infrared absorbance spectra at high speeds [6]. This allows the facile collection of hyperspectral images, where each pixel provides the corresponding spatially resolved absorption as a function of wavenumber.

## Morphological effects

The increasing availability of imaging systems now permit the analyses of non-homogeneous samples, where structural changes accompany changes in chemical composition. However, these samples introduce additional spectral characteristics due to the sample morphology. These effects can dramatically affect the ability to differentiate between chemical constituents. Morphological characteristics can affect the spectrum in two ways: (a) increased absorbance as a function of density and thickness, and (b) scattering affects. Changes in tissue thickness and density result in well-understood spectral changes characterized by the Beer-Lambert Law, while scattering is significantly more difficult to characterize.

### Scattering

Light transmitted through non-homogeneous samples is subject to scattering as it transitions between material interfaces exhibiting different indices of refraction, $n(\bar{\nu })$. These effects are prominent in mid-infrared spectroscopy, where the re-direction of light is indistinguishable from absorbance [7-9], resulting in wavelength-dependent changes in the absorbance spectrum that make the determination of the actual tissue absorbance, $A(\bar{\nu })$, extremely difficult [7]. The study of scattering effects in FT-IR imaging is an active area of research [8-10]. Current work suggests that a significant portion of scattering through tissue samples is the result of interaction with microscopic structures, such as cell bodies and nuclei [11]. Empirical methods have been proposed for correcting spectra based on Mie theory [12], however these techniques are time consuming and do not provide interactive feedback. In addition, Mie theory is not generally applicable to scattering effects and the prior information required for these estimations is not always available. While computational methods have been proposed for eliminating scattering effects for known structures such as spheres [13], no automated techniques have been proposed that compensate for spectral features introduced by elastic scattering, which accounts for a large amount of variance in mid-infrared spectroscopic images [14].

An alternative method presents a first-order approximation to remove the non-chemical effects of scattering [15]. For each feature, a corrected absorption spectrum $Â(\bar{\nu })$ is determined by subtracting a piecewise-linear function *b*:

(2) $$Â(\bar{\nu })=A(\bar{\nu })-b(\bar{\nu })$$

However, the process requires manual specification of points that represent *b* as well as either detailed knowledge of the chemical compound under consideration or extensive exploration of the data set. This makes the selection of spectral features representing chemical information extremely time-consuming, particularly when the chemical composition of the tissue sample is unknown.

### Beer-Lambert law

Variations in tissue thickness and molecular density cause linear scaling of the absorbance spectrum according to the Beer-Lambert Law:

(3) $$Â(\bar{\nu })=\kappa (\bar{\nu })\mathrm{\ell N}$$

Here, $Â(\bar{\nu })$ is the scattering-corrected absorption as a function of the wavenumber $\bar{\nu }$, $\kappa (\bar{\nu })$ is the absorption coefficient, *ℓ* is the path length through the specimen (thickness), and *N* is the molecular density. The absorption coefficient *κ* is the desired material property that defines the chemical composition of the specimen.

Note that there are no methods for separating the path length *ℓ* and density *N* in the general case. However, the function $\kappa (\bar{\nu })$ can be estimated using a reference band ${\bar{\nu }}_{r}$ within the spectrum. One example of a useful reference is the Amide I peak found in biological tissue at ${\bar{\nu }}_{r}\approx$1650cm ^−1^. By normalizing Amide I absorption across chemical species (setting $A({\bar{\nu }}_{r})=1.0$), the combined contributions of path length and density are estimated in terms of the relative protein composition, using $A({\bar{\nu }}_{r})=\mathrm{\ell N}$, and determining the normalized spectrum:

(4) $$A_{n}(\bar{\nu })=\frac{Â(\bar{\nu })}{Â({\bar{\nu }}_{r})}$$

While a single reference feature is useful, it is not generally applicable to samples containing several unique components, therefore several references are used for classifying multiple chemical species.

## Methods

In this section, we describe our methods for interactive exploration of hyperspectral data. The chemical composition of a sample is identified by finding features, such as absorbance peaks, that can be used as references and to differentiate individual compounds. For example, the biological compound *collagen* exhibits several absorbance peaks between 1235cm ^−1^ and 1265cm ^−1^. However, these peaks are difficult to distinguish in a raw spectrum, due to morphological characteristics of the sample (Section ‘Morphological effects’).

Given a measured absorption spectrum from a non-homogeneous sample composed of a single chemical constituent, Equations 2 and 4 indicate that the normalized, corrected spectrum can be computed using:

(5) $${\hat{\text{A}}}_{\text{n}}(\bar{\nu })=\frac{A(\bar{\nu })-b(\bar{\nu })}{A({\bar{\nu }}_{r})-b({\bar{\nu }}_{r})}$$

where ${\bar{\nu }}_{r}$ is the location of a spectral feature used for normalization and $b(\bar{\nu })$ is an estimate of the spectral contribution due to scattering.

For an unknown sample, both the normalization feature and baseline function must be estimated. In addition, if the sample is composed of several unique chemical constituents, multiple normalization features and baseline functions must be utilized for classification. This is an extremely difficult problem to solve computationally, requiring detailed knowledge of both the sample and the relationship between spectral bands and molecular characteristics.

The SpecVis software addresses this problem by allowing interactive visualization of the data set. Our software allows a spectroscopist to specify the baseline function *b* (Section ‘Scattering’), dynamically select reference features (Section ‘Beer-Lambert law’), and visualize the chemical characteristics. This is done by allowing the user to specify changes in the distribution of spectra, as reflected using a dynamic 2D histogram. The user then selects chemical features in this histogram, exploring the results through an interactive 2D visualization of the tissue sample. Computing the changing distribution of spectra as well as visualization of user-selected features is computationally expensive, making interactive feature selection impossible on current CPU-based desktop systems. We therefore demonstrate that this problem can be well-formulated for implementation on programmable graphics hardware. In the following sections, we first describe the types of metrics used to identify features in hyperspectral images. We then discuss how spectra in an image are adjusted to remove scattering artifacts and other distortions, based on a histogram describing the distribution of spectral information.

### Metrics

We first identify measures of spectral features, or metrics, that quantify, for example, characteristics in forensic spectroscopy [16] and differentiate cell types in biological samples by acting as features for more complex classification systems [2,17]. The user specifies parameters for these measurements in the spectral domain. Once specified, a metric is immediately applied to all pixels in the image. The metrics described in this paper include the peak height, which is the most basic measure of chemical composition, as well as peak integral and centroid. While these are not all of the possible types of metrics, we limit our study to these as this is an active area of research and they allow us to identify several important chemical differences in biological tissue samples. Additional types of metrics can be readily added as our approach is general. For each metric, the function S(x, y, $\hat{\text{nu}}$) represents a generic spectral value, where (x, y) is the spatial position and $\hat{\text{nu}}$ is the wavenumber. In the case of absorbance measurements, S can be either the raw or corrected absorbance spectrum.

#### Peak height

This metric is highly sensitive to peak shifts. This is particularly noticeable for the Amide I peak at 1650cm ^−1^, which is narrow and composed of multiple chemical contributions that make it prone to shift. Absorbance is particularly useful for detecting broad peaks and the density of well-known and localized molecular bonds.

#### Peak integral

While the peak absorbance is indicative of the presence of species, the total area under the peak is indicative of the total concentration. Hence, another useful metric is the definite integral of the spectrum on a user-specified interval $\left[{\bar{\nu }}_{0},{\bar{\nu }}_{1}\right]$ that provides the area under the peak:

(6) $$M_{i}(x,y,{\bar{\nu }}_{0},{\bar{\nu }}_{1})=\int_{{\bar{\nu }}_{0}}^{{\bar{\nu }}_{1}}S(x,y,\bar{\nu })d\bar{\nu }$$

This metric provides a robust measure of absorbance within a spectral region and is relatively insensitive to noise and peak shifts. This robustness makes it an ideal candidate for use as a spectral reference. It is insensitive, however, to subtle spectral changes that are often found in biological tissue samples.

#### Centroid

The centroid of a bounded region of the spectrum is computed using:

(7) $$M_{c}(x,y,{\bar{\nu }}_{0},{\bar{\nu }}_{1})=\frac{\int_{{\bar{\nu }}_{0}}^{{\bar{\nu }}_{1}}\bar{\nu }S(x,y,\bar{\nu })d\bar{\nu }}{\int_{{\bar{\nu }}_{0}}^{{\bar{\nu }}_{1}}S(x,y,\bar{\nu })d\bar{\nu }}$$

The resulting value is the wavenumber for the center of mass in the specified region. This metric is useful for measuring shifts in single peak positions as well as the distribution of multiple species’ absorption among multiple neighboring peaks. This metric is dependent on the distribution of absorption and therefore does not require a reference. However, it is incapable of detecting the height and is of limited utility for peaks that do not shift as a function of spatial position.

### Interactive histogram

The first step in visualization is to display the distribution of spectra in the image using a 2D histogram. This allows the user to identify chemical compounds by approximately removing morphological effects and selecting metrics. Chemical features are selected using the joint histogram of wavenumber and absorbance for all spectra in the image. The user selects features in this domain that correspond to the structural and chemical components of the tissue sample. However, the data processing required to separate structural and chemical features is currently time consuming and the results are difficult to visualize. We implement a dynamic approach, which allows the user to explore the data set via interactive feedback in both the spatial and spectral domains, which facilitates meaningful feature selection. Our framework allows the user to interactively adjust points for baseline correction and select reference features. Data processing is performed dynamically on the GPU using CUDA [18] and provides interactive feedback for multi-gigabyte data sets. The unprocessed data set is stored on the GPU as a three-dimensional texture map represented as 32-bit floating point values. The user explores the data in two ways: (a) the insertion of baseline points to build the scattering approximation $b(\bar{\nu })$ and (b) the selection of a reference metric. The histogram is computed interactively and dynamically as the user changes parameters for the baseline and reference. This is done using a CUDA device kernel. A block of threads is assigned to process each band (wavenumber). We specify a 2D block size of $\sqrt{w}\text{x}\sqrt{w}$ threads, where *w* is the maximum warp size supported by the GPU. Therefore, each block consists of one warp that executes data in a single-instruction multiple-data (SIMD) fashion. Each block *b* is responsible for computing the complete one-dimensional histogram for a single band $\bar{\nu_{b}}$. The threads within each block are responsible for evaluating a spatially coherent square of pixels, initially positioned at the upper left-hand corner of the image. Each thread then iterates across the image to the lower-right corner at intervals of $\sqrt{w}$. Note that all threads within the block are part of the same (SIMD) warp, therefore they will be spatially coherent at each iteration across the spatial domain of the image. This spatial coherence is used to perform faster fetches using texture units, which is particularly useful for fast evaluation on GPUs with compute capability lower than 2.0.

Computing the processed spectrum at each spatial location within a band requires a maximum of four memory fetches: (1) the raw data value at $A(x,y,\bar{\nu })$, (2) the raw values at the baseline points $A(x,y,{\bar{\nu }}_{0})$ and $A(x,y,{\bar{\nu }}_{1})$, and (3) the reference value *r*(*x*,*y*). Computing the reference value *A*_*r*_(*x*,*y*) also requires its neighboring baseline points at $A(x,y,\bar{nu_{r0}})$ and $A(x,y,\bar{\nu_{r1}})$. However, an image of the reference values at each spatial location is pre-computed whenever the reference is changed.

As each thread traverses the image at $\bar{\nu }$, the resulting histogram is accumulated in shared memory allocated for the block. Accurate computation of the histogram, however, requires the use of atomic operations for incrementing the counters for each bin. This can reduce performance when multiple threads encounter similar absorbance values – a common occurance, given the close spatial proximity of all threads in the $\bar{\nu }$ image. In addition, the SIMD execution within a warp will cause the entire block to pause while atomic adds are resolved. We address this issue by allocating a separate shared histogram for each thread and summing the results when the entire $\bar{\nu }$ image has been processed. The resulting histogram is then displayed using a *log*-scale intensity filter.

### Visualization

The chemical composition of the tissue sample is visualized by building a 2D image based on user-selected metrics. Each metric is assigned a color value, where the intensity is based on the value of the metric. The metrics are then evaluated for each pixel, and the resulting colors are combined to create a spatially-resolved chemical visualization of the sample. This color-mapping technique is similar to a *transfer function*, which is commonly used in volumetric visualization. We first provide an overview of this technique and describe how it is applied to our algorithm.

#### Transfer functions

Transfer functions are used in volume visualization to assign color and opacity values to pixels based on features defined in a separate domain [19,20]. These techniques generally use spatial features such as gradient magnitude [21], curvature [22], size [23], and orientation [24] to assign color values. While these techniques have been applied to gigabyte-scale data sets [25,26], they are difficult to generalize to spectroscopic images since each pixel represents a spatially-resolved absorbance function. The principle behind using spectra to apply transfer functions has been previously explored through contour spectra [27], where spatial characteristics are used to highlight geometric features in the data set. More flexible methods using spatially local statistics have also been proposed [28]. Both contour spectra and spatial statistics may be applicable for extracting spatial characteristics in heterogeneous samples. In this paper, we focus on visualizing chemical features, and therefore each pixel is considered to be an independent component with a corresponding chemical signature.

Very few techniques currently exist for visualizing spectroscopic images. Li et al. [29] propose a technique for visualizing astrophysical data imaged at various wavelengths. They propose the use of transfer functions for defining opacity when rendering the image stack volumetrically. However, the number of bands in a single image is small and the samples are discontinuous, which limits the amount of chemical information in the data set. More recent work demonstrates a visualization framework for near-infrared spectroscopic images of historical documents sampled regularly in the spectral domain [30]. This technique allows relighting of images, interactive selection of individual bands, and metrics for evaluating similarity between user-specified spectra. However, similarity is difficult to measure for vibrational spectra because of the coupled structural and chemical contributions to the spectrum. Unsupervised techniques, such as principal component analysis (PCA) and vertex component analysis (VCA) [31], have been proposed to perform spectral unmixing. However, these techniques assume homogeneous samples and also make specific assumptions about the data, such as the existence of orthogonal chemical signatures, which are not generally applicable. Comprehensive Data Maps (CDM) have been proposed to examine data [32], but do not provide imaging visualizations.

**Figure 2:**
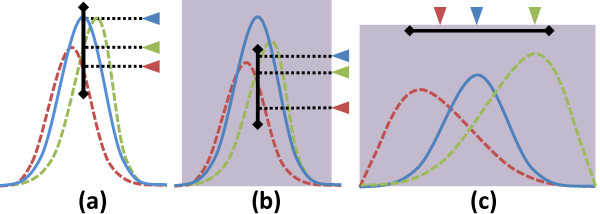
**Metrics for selecting spectral features.** Useful measurements include (**a**) peak height, (**b**) average peak height (integral), (**c**) centroid (center of gravity). Colored arrows indicate the projection of the metric value on the widget (black line). Shaded regions indicate the integral intervals.

**Figure 3:**
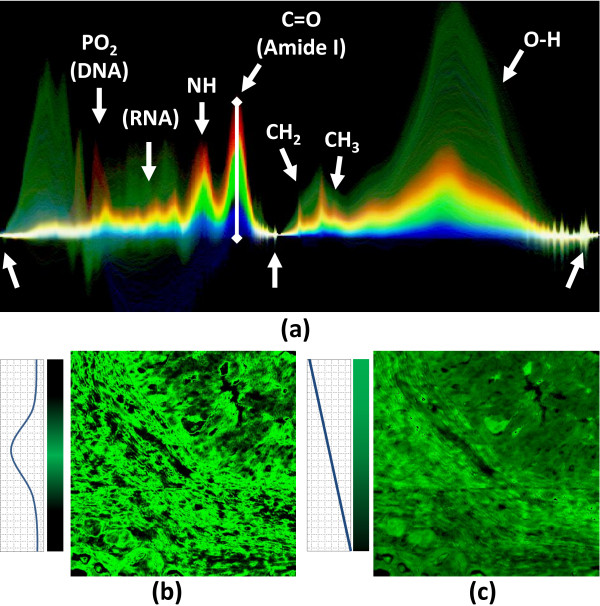
**Color mapping of spectral features in a baseline-corrected image.** Labels indicate peaks representing prominent chemical features. (**a**) The spectral histogram shows a rainbow color map applied based on the peak height of the Amide I band (vertical white line). The peak height of Amide I is often assumed to correspond to tissue thickness and density in tissue samples. Note how the colors mix as individual spectra cross and overlap with changes in density and chemistry. Spatial images show the result of a (**b**) Gaussian and (**c**) linear ramp applied to the lasso.

Color values are assigned to spectra based on metric results. The user specifies a bounding range for a metric value using a lasso tool in the spectral histogram. For all three metrics, the lasso bounds are indicated by a line segment. For metrics that are computed across a specified wavenumber interval, a shaded region highlights these boundaries (Figure 2). When the result of a spectral metric falls within these bounds, a color value is assigned based on the point of intersection using a ramp shader. Our framework allows the user to assign colors using a constant, linear, and Gaussian ramp (Figure 3), as available in most image analysis software. In order to make selection intuitive for the integration metric *M*_*i*_, color is assigned based on the average peak height

(8) $${\tilde{M}}_{i}=\frac{M_{i}}{{\bar{\nu }}_{1}-{\bar{\nu }}_{0}}$$

rather than the integral. This allows the widget to be placed in the vicinity of the selected spectra.

#### Computing metrics

**Figure 4:**
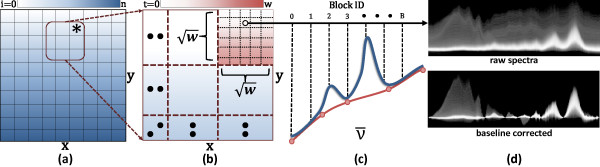
**Parallel implementation for evaluating the corrected spectral distribution.** Each band is evaluated by a block of $\sqrt{w}\text{x}\sqrt{w}$ threads. (**a**) Each block iterates across the spatial dimensions of the image from *i*=0 to *i*=*n*, where $n=\frac{S_{x}S_{y}}{w}$ and *S*_*x*_ and *S*_*y*_ are the spatial extents. The threads for *i*=14 (*) are shown. (**b**) The threads are evaluated using SIMD and kept spatially coherent to take advantage of 2D texture caching. (**c**) Each band is evaluated by an independent thread block and each thread performs a maximum of 3 texture fetches per iteration (1 data point and 2 baseline points). (**d**) Resulting histogram.

Metrics are computed independently for each spectrum, requiring the appropriate baseline points and reference bands. The data set is partitioned spatially into $\sqrt{w}\text{x}\sqrt{w}$ blocks, where each thread is assigned an independent spectrum. The baseline bands and reference are applied using a GPU kernel (Figure 4). If the metric requires integration, summation occurs along the spectral axis. Note that a binary search for the neighboring baseline bands is only necessary when a thread is created. If a baseline band is crossed within an interval of integration, the next band ${\bar{\nu }}_{n}$ in the list is acquired. The metric result is saved to a 2D grid, which can be used as a source for color-mapping to produce the final display image or as a reference for additional metrics.

## Results and discussion

Rendering time is dominated by the computation of the histogram as the user changes processing and visualization parameters. However, the frame rate is interactive for our largest sample image (700x700x491, ≈1GB), requiring <32ms for complete evaluation of the histogram (<147ms with atomic writes to shared memory) using a GeForce GTX 580 with 1.5GB of global memory. We use the developed software to identify characteristics in mid-infrared spectroscopic images. We first demonstrate the visualization of structural and chemical components from images of synthetic polymer targets that are often used to assess image quality. We then show how these techniques can be used to extract similar information from mid-infrared images of tissue biopsies, including the visualization of chemical components useful for breast cancer diagnosis. Finally, we demonstrate that these techniques can be extended to other forms of spectroscopy by visualizing Raman images of tissue samples.

### Infrared images of tissue

**Figure 5:**
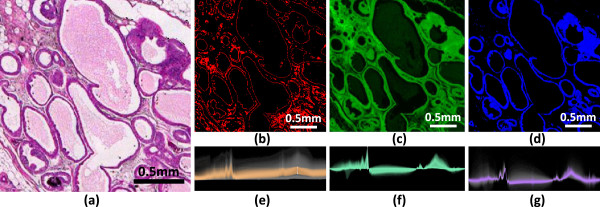
**Using transfer functions to visualize features seen in standard histology.** A surgical resection of a breast biopsy was imaged using IR spectroscopy and then stained and imaged with a standard optical microscope. (**a**) A section of the hematoxylin and eosin (H&E) stained tissue is shown. The proposed techniques can be used to selectively visualize various structural and chemical features using only the IR hyperspectral image. (**b**) Scattering effects are highlighted, indicating regions containing highly scattering structures (edges and collagen fibers). (**c**) Removal of scattering features allows the selection of tissue thickness based on the absorbance of Amide I (1650cm ^−1^). (**d**) Using Amide I as a reference, chemical compounds corresponding to epithelial cells are visualized using the centroid of the Amide II band (≈1550cm ^−1^). (**e-f**) The corresponding joint histograms are shown, with selected pixels highlighted.

Biological tissue samples pose a unique problem in spectroscopic imaging, since each pixel contains a complex combination of chemical species. Spectral features that correspond to different tissue types are subtle and difficult to identify, making interactive exploration a valuable tool. We first demonstrate the structural and chemical characteristics that can be visualized using the developed techniques. We obtain a tissue sample and place it on a barium fluoride substrate for imaging. The same sample is then stained using a commonly-used clinical dye, Hematoxylin and Eosin (H&E), in order to identify structural and chemical features (Figure 5a).

**Figure 6:**
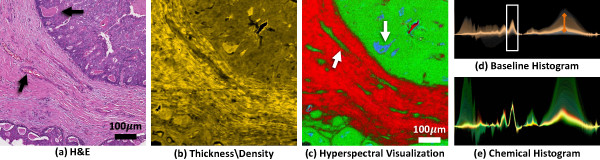
**Visualization of a 1mm needle biopsy from a breast tissue biopsy at 74X magnification (scale bar = 100*μ*m).** (**a**) An adjacent histology slide stained with H&E is shown. Epithelial cells are purple and stromal cells are pink. Arrows indicate the position of a blood vessel and necrosis. (**b**) Tissue density is shown after baseline correction. (**c**) Features are then selected to separate stromal cells (red), epithelium (green), and necrosis (blue). The histogram shows selected spectra for estimating tissue density (**d**) and chemical composition (**e**). The composition histogram is normalized to the area under the Amide I peak (white box).

The developed method is used to find similar features from the hyperspectral image data. Since the unprocessed spectra are dominated by scattering in the high-wavenumber regions, we are able to visualize pixels containing edges and boundaries, where scattering is more prominent (Figure 5b and e). We then select baseline points and use a linear gradient to visualize the tissue thickness and density based on Amide I absorption (Figure 5c and f). Using Amide I as a reference, features in the spectra are now dominated by chemical differences between cell types in the tissue. This allows the user to visualize the distribution of tissue types, such as epithelial cells (Figure 5d and g), in which 97% of cancers of the breast arise. The facile delineation of epithelial cells is a desirable, but unmet need, as reported in recent studies using automated computerized analysis of pathologic images [33]. We further demonstrate the possible use of the presented techniques for tissue pathology. Several breast cancer biopsies were commercially obtained and stained using H&E. Adjacent tissue samples were imaged using mid-infrared spectroscopy. We then designed transfer functions to identify tissue characteristics that are useful for cancer diagnosis. This includes structural features, such as tissue density, as well as labeling of various tissue types. These types include epithelial cells, connective tissue (stroma), and necrotic (dead and dying) cells (Figure 6). The spatial distribution of these features are commonly used in pathology to identify the presence of a tumor.

**Figure 7:**
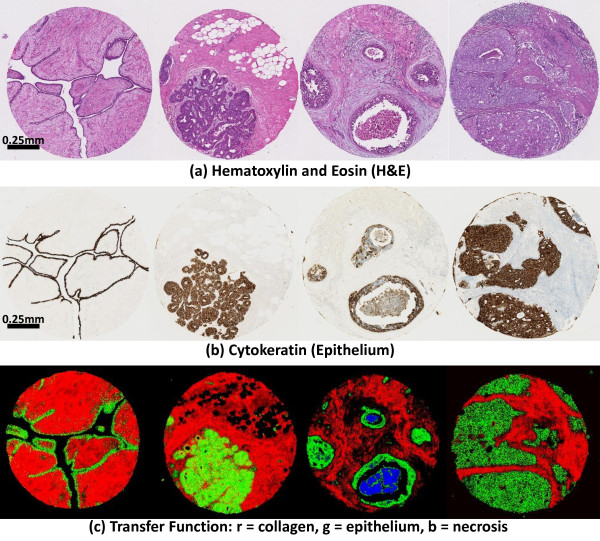
**Standard histology stains (top, middle) and hyperspectral imaging (bottom) applied to adjacent sections of 1mm breast cancer biopsies.** Hyperspectral imaging is quantitative and our methods allow the pathologist to separate several chemical constituents consistently across tissue samples. Adjacent sections of 1mm biopsy cores are shown stained using hematoxylin and eosin (**a**), cytokeratin for epithelium (**b**), and visualized using our methods (**c**). The SpecVis visualization shows epithelial cells in green, collagen in red, and necrosis in blue. Separating epithelium and stromal cells in H&E stained tissue can be difficult (**a**), however these differences are visible using a cytokeratin stain (**b**) for epithelium. Necrosis is difficult to identify using chemical staining (**a** and **b**), but can be clearly segmented using mid-IR spectroscopic imaging combined with our processing methods (**c**, blue).

Once the transfer function is created, it is then applied to other tissue samples (Figure 7). Note that these tissue types are difficult to separate using chemical staining. Often, mutiple stains are necessary for diagnosis and these methods are difficult to quality-control. However spectroscopic imaging provides quantitative data that can be used to characterize chemical information reliably across multiple samples. The ability to identify tissue types by interactively exploring the hyperspectral data provides several advantages over existing techniques. Unlike chemical staining, mid-infrared imaging does not perturb the tissue sample in any way. In addition, the effects of scattering on tissue samples are not well understood, and previous methods for classification of tissue types require extensive pre-processing and the application of machine learning [2,34]. Though an extensive comparison between the more complex machine learning methods and the method reported here must be undertaken to quantify advantages, the results seem to be comparable and significantly faster for our method by facilitating interactive analysis. An experienced user was able to separate, visualize, and evaluate the utility of the extracted tissue characteristics useful for tumor diagnosis in a few minutes, given interactive feedback. Using our older methods, simply computing a metric took several minutes on a CPU.

### Raman spectroscopy

**Figure 8:**
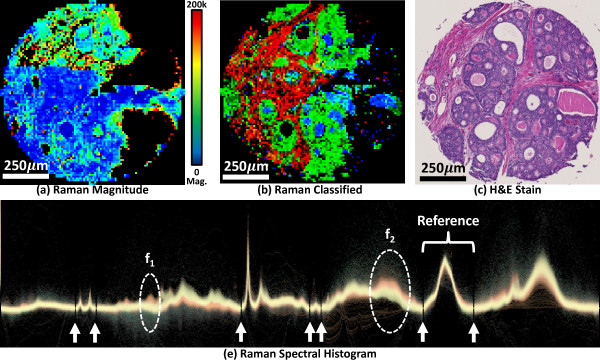
**Application of hyperspectral transfer functions on Raman spectroscopy.** A single breast tumor biopsy was imaged using Raman microscope. The magnitude of the Raman signal is shown using a rainbow color map (**a**). A new transfer function is applied to separate cell types (**b**) based on the adjacent histology (**c**). Colors reflect stroma (red), epithelium (green), and protein deposits (blue). The Raman spectral distribution is shown (**e**) with features and baseline points indicated.

The methods that we propose in this paper can also be extended to hyperspectral imaging in other areas. We demonstrate that transfer functions can be created to effectively visualize the distribution of chemical compounds in Raman images. We collected Raman images of prostate biopsy cores and built a transfer function to discriminate between epithelial and stromal cells (Figure 8). Some advantages of Raman spectroscopy include the ability to choose the excitation frequency, easy coupling to fiber-optic probes, and little to no signal from water in a sample. These features make it useful for taking *in vivo* measurements [35-37]. However, the Raman spectral signal is extremely weak and requires long acquisition times that effectively limit the signal-to-noise ratio (SNR) and the size of image that can be obtained in a reasonable amount of time. Our method is robust and can thus be generally applied.

## Conclusions

The ability to acquire spatially resolved information using hyperspectral imaging is strongly emerging as a promising avenue across a variety of areas, especially biomedical analyses. Vibrational spectroscopy has the potential to provide quantitative histology for disease diagnosis, without the use of chemical stains in an objective and automated manner. Recent work has shown that mid-infrared spectroscopic imaging provides sufficient chemical detail for differentiating between tissue types. Researchers have reported very high accuracy after rigorous scattering correction [12] and classification algorithms [2,3] are applied. These studies demonstrate the potential for applying vibrational spectroscopy to cancer diagnosis.

However, computational methods must be developed to visualize the data quickly and reliably. The size and complexity of the data contained in hyperspectral images makes this a difficult problem, requiring the separation of physical and chemical characteristics from underlying spectra. In this paper, we demonstrate an interactive method for building transfer functions for visualizing hyperspectral images. Our method allows users to dynamically assimilate large collections of spectra using algorithms designed to separate structural and chemical features in real time. These features are then selected using transfer functions which allow the visualization of these characteristics in a spatial image of the sample. To our knowledge, the reported methods are the first to allow interactive processing and visualization of hyperspectral images at this level of spectral and structural detail, and we have demonstrated their usefulness in biological samples. Applying these features to tissue provides a method for label-free identification of tissue types that is quantitative, non-destructive, and can be performed in a time frame that is clinically viable.

Future directions include applying these techniques to three-dimensional samples, which can be acquired using Raman spectroscopy in combination with a confocal microscope, for example.

Our proposed technique also has several advantages over unsupervised methods, such as PCA and VCA. In particular, the metrics that we use for visualization have finite support, requiring only a narrow band of information within the spectrum. Once useful metrics are identified, the number of collected bands can then be reduced, allowing faster imaging, for example using narrow-band filters for IR [38]. Finally, since the separation of structural and chemical characteristics from an IR image is so difficult, many algorithms for the classification of hyperspectral images rely on the use of user-defined metrics [2]. Our method may provide an efficient method for selecting features for use in more complex classifiers for IR-based clinical histology.

## Competing interests

The authors declare no competing interests.

## Authors’ contributions

DM designed the algorithm and developed the software. MW collected mid-infrared images and identified spectral and chemical features corresponding to cell types. MS collected Raman images and identified spectral and chemical features corresponding to cell types. RB designed the experiments and identified chemical and spectral features corresponding to cell types in cancer biopsies. All authors read and approved the final manuscript.
